# P-482. Colonization and Infection with Carbapenem-Resistant Organisms in Critically Ill Neonates: A Prospective Cohort Study in a Tertiary Hospital in Bangladesh

**DOI:** 10.1093/ofid/ofaf695.697

**Published:** 2026-01-11

**Authors:** Fahmida Chowdhury, Gazi Md Salahuddin Mamun, Sanzida Khan, Md Aminul Islam, Dilruba Ahmed, Aninda Rahman, Shabrina Sharmin, Md Shahinur Rahaman, Tahsinul Amin, Syeda Mah-E-Muneer, Gemma Parra, Ashley R Styczynski

**Affiliations:** icddr,b, Dhaka, Dhaka, Bangladesh; International Centre for Diarrhoeal Disease Research, Bangladesh (icddr,b), Dhaka, Dhaka, Bangladesh; icddr,b, Dhaka, Dhaka, Bangladesh; icddr,b, Dhaka, Dhaka, Bangladesh; icddr,b, Dhaka, Dhaka, Bangladesh; Directorate General of Health Services, Government of Bangladesh., Dhaka, Dhaka, Bangladesh; International Centre for Diarrheal Disease Research, Bangladesh, Dhaka, Dhaka, Bangladesh; International Centre for Diarrheal Disease Research, Bangladesh, Dhaka, Dhaka, Bangladesh; Dhaka Medical College and Hospital, Dhaka, Dhaka, Bangladesh; icddr,b, Dhaka, Dhaka, Bangladesh; Centers for Disease Control and Prevention, Atlanta, GA, United States, Atlanta, Georgia; Centers for Disease Control and Prevention, Atlanta, GA

## Abstract

**Background:**

Antimicrobial resistance is an increasing cause of neonatal infection-related mortality. Colonization with carbapenem-resistant organisms (CRO) may precede and increase the risk for subsequent infection in neonates. In this study, we evaluated associations between CRO colonization and infectious outcomes among neonates admitted to a neonatal intensive care unit (NICU).

**Figure:**
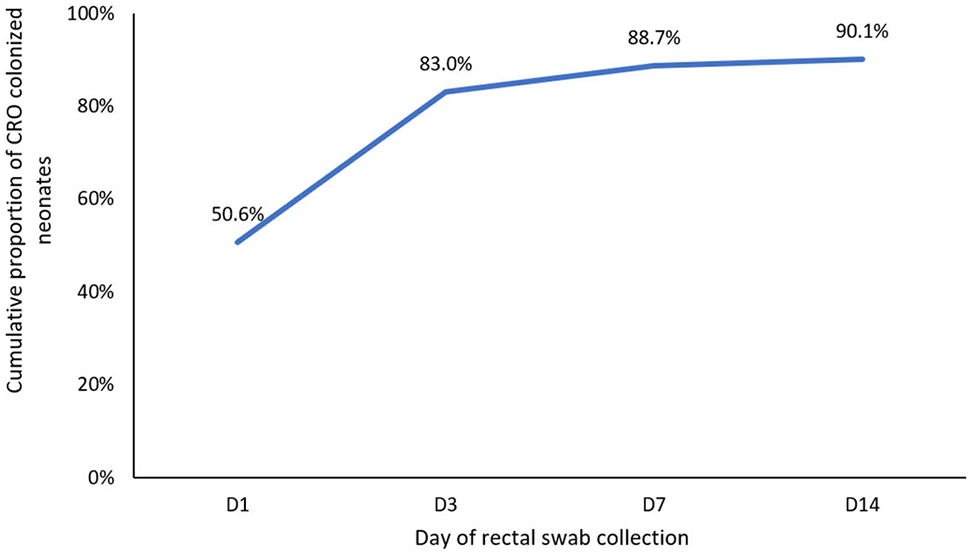

**Figure:**
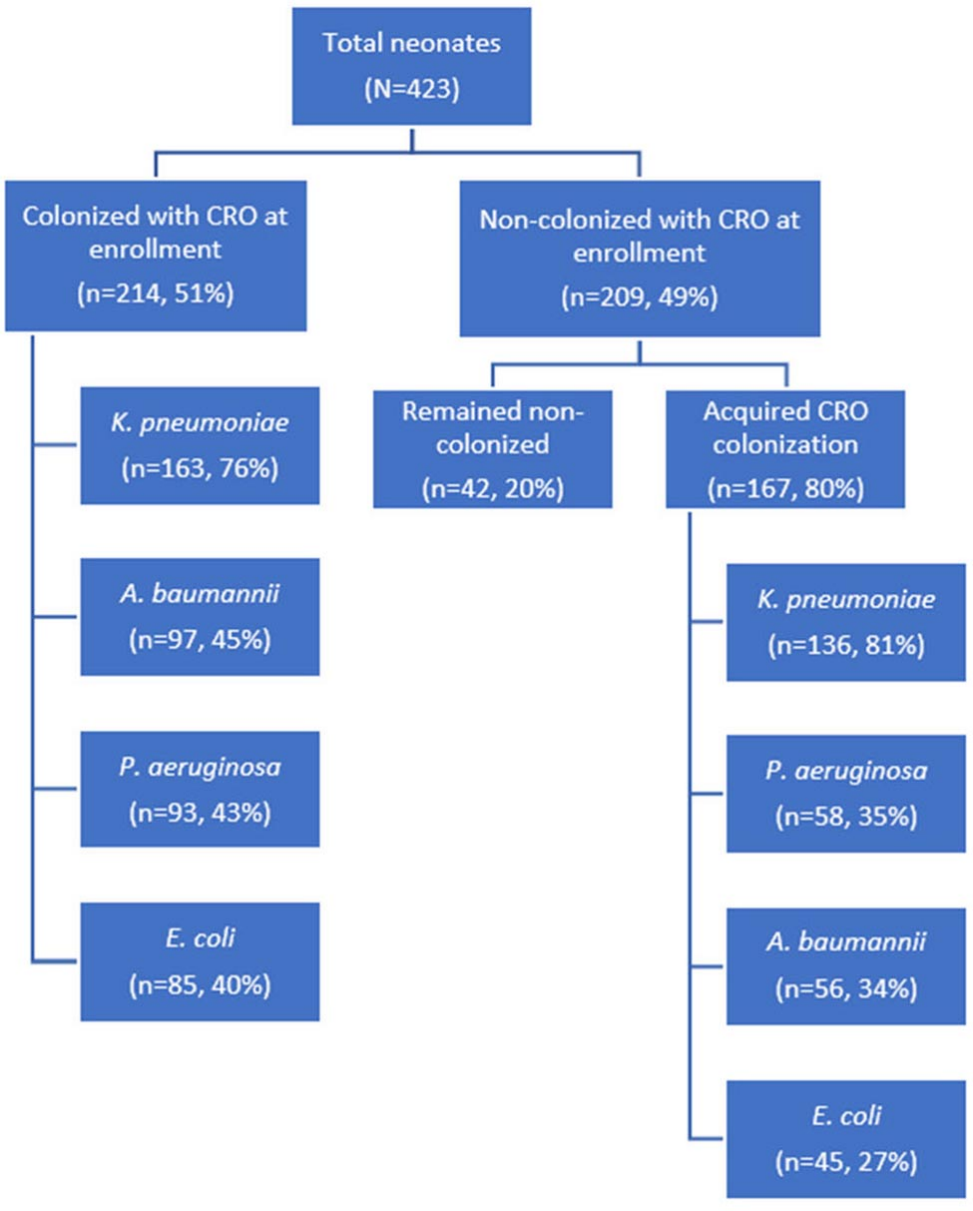

**Methods:**

From July 2023 to February 2024, a prospective cohort study was conducted among neonates admitted to a tertiary hospital NICU. Neonates were assessed for CRO colonization using rectal swabs collected within 24 hours of admission, on days 3 and 7, and weekly until discharge, transfer, or death. Swabs were plated on selective agar followed by VITEK-2 identification and susceptibility testing. VITEK-2 was also used for blood and tracheal aspirate culture isolates in suspected sepsis cases. Risk ratios (RR) and other analyses were performed using Stata v15.

**Results:**

From July 2023 to February 2024, 423 neonates were enrolled (median age 4 days; IQR 2–8), 62% male. At enrollment, 51% (214) were colonized with CROs. Of those who were not colonized at enrollment, 80% (167/209) acquired colonization during their hospital stay (Figure 1). Among CRO-colonized neonates, 71% had *Klebsiella pneumoniae* (*Kpn*), 36% *Pseudomonas aeruginosa*, 36% *Acinetobacter baumannii*, and 31% *Escherichia coli* (Figure 2). Clinically suspected infections occurred in 113 (27%) neonates. Of 33 positive bacterial cultures, 10 (30%) were CROs, including *Kpn* (4), *E. meningoseptica* (2), *A. baumannii* (1), *A. lwoffii* (1), *E. coli* (1), and *B. cepacia* (1). Three clinical isolates had antibiotic susceptibility patterns matching with colonized strains; all were *CR-Kpn*. CRO colonization at any time point was associated with confirmed bacterial infection (RR 1.11, 95% CI 1.05–1.17).

**Conclusion:**

CRO colonization was common among NICU patients and increased throughout the hospital stay. These findings highlight the urgent need of targeted prevention strategies to reduce CRO transmission and infection risk among this vulnerable population.

**Disclosures:**

All Authors: No reported disclosures

